# Response to Hypoxia and the Ensuing Dysregulation of Inflammation Impacts *Mycobacterium tuberculosis* Pathogenicity

**DOI:** 10.1164/rccm.202112-2747OC

**Published:** 2022-04-12

**Authors:** Allison N. Bucşan, Ashley Veatch, Dhiraj K. Singh, Sadia Akter, Nadia A. Golden, Melanie Kirkpatrick, Breanna Threeton, Chivonne Moodley, Mushtaq Ahmed, Lara A. Doyle, Kasi Russell-Lodrigue, Elizabeth B. Norton, Peter J. Didier, Chad J. Roy, Robert B. Abramovitch, Smriti Mehra, Shabaana A. Khader, Deepak Kaushal

**Affiliations:** ^1^ Tulane National Primate Research Center, Tulane University Health Sciences Center, Covington, Louisiana;; ^2^ Southwest National Primate Research Center, Texas Biomedical Research Institute, San Antonio, Texas;; ^3^ Department of Molecular Microbiology, Washington University in St. Louis School of Medicine, St. Louis, Missouri;; ^4^ Department of Microbiology and Immunology, Tulane University School of Medicine, New Orleans, Louisiana; and; ^5^ Department of Microbiology and Molecular Genetics, Michigan State University, East Lansing, Michigan

**Keywords:** mycobacterium, tuberculosis, hypoxia

## Abstract

**Rationale:**

Different *Mycobacterium tuberculosis* (*Mtb*) strains exhibit variable degrees of virulence in humans and animal models. Differing stress response strategies used by different strains of *Mtb* could influence virulence.

**Objectives:**

We compared the virulence of two strains of *Mtb* with use in animal model research: CDC1551 and Erdman.

**Methods:**

Rhesus macaques, which develop human-like tuberculosis attributes and pathology, were infected with a high dose of either strain via aerosol, and virulence was compared by bacterial burden and pathology.

**Measurements and Main Results:**

Infection with Erdman resulted in significantly shorter times to euthanasia and higher bacterial burdens and greater systemic inflammation and lung pathology relative to those infected with CDC1551. Macaques infected with Erdman also exhibited significantly higher early inflammatory myeloid cell influx to the lung, greater macrophage and T cell activity, and higher expression of lung remodeling (extracellular matrix) genes, consistent with greater pathology. Expression of NOTCH4 (neurogenic locus notch homolog 4) signaling, which is induced in response to hypoxia and promotes undifferentiated cellular state, was also higher in Erdman-infected lungs. The granulomas generated by Erdman, and not CDC1551, infection appeared to have larger regions of necrosis, which is strongly associated with hypoxia. To better understand the mechanisms of differential hypoxia induction by these strains, we subjected both to hypoxia *in vitro*. Erdman induced higher concentrations of DosR regulon relative to CDC1551. The DosR regulon is the global regulator of response to hypoxia in *Mtb* and critical for its persistence in granulomas.

**Conclusions:**

Our results show that the response to hypoxia is a critical mediator of virulence determination in *Mtb*, with potential impacts on bacillary persistence, reactivation, and efficiency of therapeutics.

**At a Glance Commentary**

**Scientific Knowledge on the Subject**

Few large studies have been performed to explore the difference in pathological outcomes in nonhuman primate models, to study infection with different strains of *Mycobacterium tuberculosis (Mtb)* in a fashion that allows for the identification of bacterial determinants that drive virulence.

**What This Study Adds to the Field**

Analyses of different strains of a pathogen is critical to identify heterogeneity in its ability to replicate, persist, and avoid host immune responses can drive virulence in host species. By utilizing multiple techniques to analyze early host immune responses, we found that rhesus macaques infected with *Mtb* Erdman had significantly higher early inflammatory myeloid cell influx to the lung, greater macrophage and T cell activity, and high expression of lung remodeling genes that supported the greater pathology and higher bacterial burdens observed in the Erdmaninfected cohort. By observing that the lungs of rhesus macaques infected with *Mtb* Erdman had more lesions and granulomas, we were able to make the connection that the increases in areas of necrosis were associated with increases in NOTCH4 signaling that are induced in response to hypoxia and promote undifferentiated cellular states. To better understand the ways that the bacterial strain was inducing or responding to hypoxia *in vivo*, we subjected bacteria to hypoxia *in vitro* and verified that *Mtb* Erdman induces higher concentrations of DosR regulon. By identifying the response to hypoxia as a major driver of virulence, additional therapeutics could be developed to manage tuberculosis in human patients.

Tuberculosis (TB) remains one of the most important infectious diseases of humanity. Of particular interest is the development of different clades and strains of *Mycobacterium tuberculosis* (*Mtb*) globally, some of which (e.g., East Asian or Beijing, lineage) appear to be more virulent than others (1). Such strains have a greater propensity to develop drug resistance (2), are more transmissible (3), and likely evade vaccine-induced protection at a significantly higher rate than other *Mtb* strains. The heterogeneity of the strains within the *Mtb* complex may contribute to the variation in the outcome of infection at the amount of both the individual and the tubercle granuloma. Despite growing evidence to this effect, we poorly understand the mechanisms by which these different strains may exhibit greater virulence.

The use of different strains of *Mtb* could allow us to better understand the mechanisms by which hosts improve protection against pathogens. Some animal models have been used for side-by-side susceptibility comparisons of different strains, such as *Mtb* Erdman, H37Rv, and CDC1551, including mice (4–7), rabbits (8, 9), and nonhuman primates (NHPs) (10). Early mouse studies failed to show differential susceptibilities between different strains of *Mtb* in intravenous or aerosol challenge (5). CDC1551 induced a more rapid and robust response in mice, with granulomas containing high concentrations of TNF-α (tumor necrosis factor-α), IL-6, IL-10, IL-12, and IFN-γ mRNA (6). A study involving comparisons of rabbits aerosol challenged with Erdman, CDC1551, and H37Rv found that Erdman was the most virulent strain (8). However, such studies have not been conducted in macaques, which are important for preclinical TB vaccine testing (11–13). Macaque studies have primarily used two *Mtb* strains: CDC1551 and Erdman. We therefore sought to compare the virulence of the two strains in the susceptible rhesus macaques. We found that Erdman is more pathogenic than CDC1551 and that the differential virulence of these two *Mtb* strains can be attributed to their differential ability to sense and respond to hypoxia. These results have important ramifications for the understanding of *Mtb*-induced necrosis, hypoxia, and inflammatory responses as well as heterogeneous responses to this infection.

## Methods

### NHP Infection and Sampling

Procedures were performed in accordance with NIH guidelines and approved by the Tulane Institutional Animal Care and Use Committee. Specific pathogen-free, mycobacteria-naive, Indian rhesus macaques aged 3–12 years, bred and housed at the Tulane National Primate Research Center were aerosol infected with ∼200 cfu of *Mtb* CDC1551 (*n* = 8) or *Mtb* Erdman (*n* = 6) (13–15). Tuberculin skin tests were performed 1–3 weeks before infection and at Weeks 3 and 5 after *Mtb* infection, as described (13, 16). Chest X-rays were performed 1 week before *Mtb* infection and at Weeks 4 and 6 after TB infection and were scored in a blinded manner by veterinary clinicians on a subjective scale of 0–4, as described (13, 17). BAL samples were collected 1 week before *Mtb* infection and every 2 weeks thereafter, as previously described, for cellular analysis by flow cytometry using well-characterized panels (17–22). Blood samples were collected 1 week before *Mtb* infection and every subsequent week to measure complete blood count and serum chemistry, including serum C-reactive protein (CRP), and for flow cytometry (15, 17, 23). Animals were necropsied once they reached a humane endpoint or 11 weeks after TB infection using predefined criteria for euthanasia as a measure for reduction of discomfort (13). Tissues were collected and processed, as previously described (13, 17). Lung pathology was evaluated in a blinded fashion, as previously described (15, 17, 23).

### Immune Analysis

Flow cytometry was performed on whole blood, polymorphonuclear blood cells (peripheral blood mononuclear cells), and BAL cells from all animals, and data were analyzed using FlowJo, as described (13, 15, 17, 19, 21, 24). At necropsy, stimulations of lung cells were performed using 10 μg/ml of H37Rv *Mtb* cell wall (BEI Resources), 50 ng/ml of phorbol 12-myristate 13-acetate (Sigma-Aldrich), and 1 μg/ml of ionomycin (Sigma-Aldrich), as previously described (20). All antibodies used in flow cytometry are shown in Table E1 in the online supplement. Cytokines were analyzed using the 37-Plex NHP ProcartaPlex Panel (Invitrogen) per manufacturer’s instructions.

### RNAseq

RNAseq on lung samples from animals infected with *Mtb* CDC1551 (*n* = 3) or Erdman (*n* = 4) was performed (Tables 1–3) as described earlier (25). DESeq2 was used to identify the significantly differentially expressed genes (DEGs) with default settings and both the *P* value and false discovery rate threshold of 0.05.

**Table 1.** Mapping Rates

Sample Name	Strain	Overall Alignment Rate (*%*)
SRR8609170	CDC1551	87.28
SRR8609176	CDC1551	79.63
SRR8609204	CDC1551	81.92
SRR8609192	Erdman	90.95
SRR8609195	Erdman	90.32
SRR8609205	Erdman	84.06
SRR8609206	Erdman	92.08

**Table 2.** Significantly Enriched Reactome Pathways among the 36 Differentially Expressed Genes That Are Overexpressed in Erdman Compared with CDC1551 Strain

ID	Description	No. of Overexpressed Genes	Total Genes	*Q* Value
R-HSA-1474244	Extracellular matrix organization	11	301	2.08E-09
R-HSA-216083	Integrin cell surface interactions	5	85	6.02E-05
R-HSA-1474228	Degradation of the extracellular matrix	5	140	0.00046812
R-HSA-3000178	ECM proteoglycans	4	76	0.00058108
R-HSA-3000170	Syndecan interactions	3	27	0.00058108
R-HSA-114608	Platelet degranulation	4	129	0.00316864
R-HSA-76005	Response to elevated platelet cytosolic Ca^2+^	4	134	0.00316864
R-HSA-9013700	NOTCH4 activation and transmission of signal to the nucleus	2	11	0.00325511
R-HSA-3000171	Non-integrin membrane–ECM interactions	3	59	0.00339237
R-HSA-2022090	Assembly of collagen fibrils and other multimeric structures	3	61	0.00339237
R-HSA-3906995	Diseases associated with O-glycosylation of proteins	3	68	0.00424058
R-HSA-400253	Circadian clock	3	70	0.00424058
R-HSA-2243919	Crosslinking of collagen fibrils	2	18	0.00551682
R-HSA-9013694	Signaling by NOTCH4	3	82	0.0057728
R-HSA-9013695	NOTCH4 intracellular domain regulates transcription	2	20	0.00592052
R-HSA-1474290	Collagen formation	3	90	0.00661871
R-HSA-6785807	IL-4 and IL-13 signaling	3	108	0.0105297
R-HSA-3000157	Laminin interactions	2	30	0.01113532
R-HSA-352230	Amino acid transport across the plasma membrane	2	33	0.01274983
R-HSA-76002	Platelet activation, signaling, and aggregation	4	263	0.0137351
R-HSA-5083635	Defective B3GALTL causes Peters-plus syndrome (PpS)	2	37	0.01446762
R-HSA-5173214	O-glycosylation of TSR domain-containing proteins	2	38	0.01455642
R-HSA-3781865	Diseases of glycosylation	3	143	0.01723865
R-HSA-1566948	Elastic fiber formation	2	45	0.01860399
R-HSA-186797	Signaling by PDGF	2	58	0.02927282

*Definition of abbreviations*: B3GALTL = β-1,3-glucosyltransferase; ECM = extracellular matrix; NOTCH4 = neurogenic locus notch homolog 4; PDGF = platelet-derived growth factor; TSR = thrombospondin type 1 repeat.

**Table 3.** Significantly Enriched Reactome Pathways among the 16 Differentially Expressed Genes That Are Overexpressed in the CDC1551 Compared with the Erdman Strain

ID	Description	No. of Overexpressed Genes	Total Genes	*Q* Value
R-HSA-2173782	Binding and uptake of ligands by scavenger receptors	3	42	0.00077284
R-HSA-1237044	Erythrocytes take up carbon dioxide and release oxygen	2	13	0.00119663
R-HSA-1480926	O_2_ and CO_2_ exchange in erythrocytes	2	13	0.00119663
R-HSA-2168880	Scavenging of heme from plasma	2	13	0.00119663
R-HSA-163560	Triglyceride catabolism	2	24	0.00335965
R-HSA-8856688	Golgi-to-ER retrograde transport	3	133	0.00400868
R-HSA-5694530	Cargo concentration in the ER	2	33	0.00446033
R-HSA-199977	ER to Golgi anterograde transport	3	154	0.00446033
R-HSA-8979227	Triglyceride metabolism	2	37	0.00446033
R-HSA-948021	Transport to the Golgi and subsequent modification	3	185	0.00625777
R-HSA-6811442	Intra-Golgi and retrograde Golgi-to-ER traffic	3	202	0.0073189
R-HSA-204005	COPII-mediated vesicle transport	2	68	0.01118028
R-HSA-381340	Transcriptional regulation of white adipocyte differentiation	2	84	0.01560549
R-HSA-446203	Asparagine N-linked glycosylation	3	304	0.01813661
R-HSA-6811434	COPI-dependent Golgi-to-ER retrograde traffic	2	99	0.01813661
R-HSA-6807878	COPI-mediated anterograde transport	2	101	0.01813661

*Definition of abbreviations*: COPI and II = coat protein complex 1 and 2; ER = endoplasmic reticulum.

### *In Vitro* Model of Hypoxia

CDC1551 and Erdman were subjected to *in vitro* hypoxia and reaeration, as described earlier (26).

## Results

### Erdman Is More Virulent Than CDC1551 in the Rhesus Macaque Model of TB

**Figure 1.**
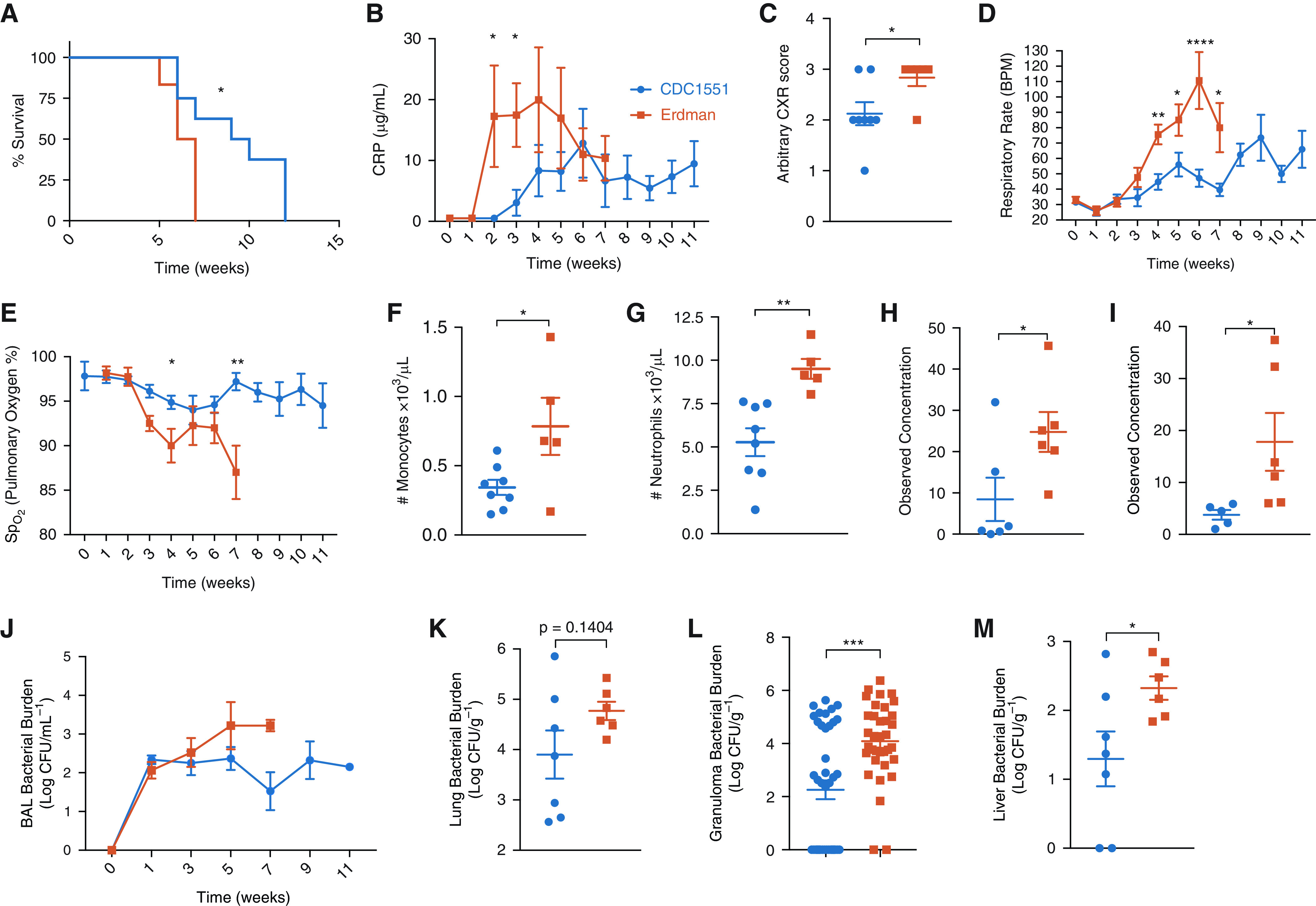
Comparative infection of rhesus macaques with *Mycobacterium tuberculosis* (*Mtb*) CDC1551 and Erdman strains. (*A*) A comparison of different *Mtb* strains shows that *Mtb* Erdman results in reduced survival compared with *Mtb* CDC1551 infection in Indian rhesus macaques with *Mtb* Erdman (*n* = 6, shown in vermillion) compared with *Mtb* CDC1551 (*n* = 8, shown in blue) (*P* = 0.0281). Log-rank (Mantel-Cox) test was used. (*B*) All animals were monitored longitudinally for clinical signs of disease, including serum (CRP); unpaired *t* test (*P* = 0.0336) was used. (*C*) CXR score was established by veterinary clinicians for pneumonia; unpaired *t* test (*P* = 0.0361). (*D* and *E*) Throughout the study, animals were monitored for signs of respiratory distress, including respiratory rate (*D*) and pulmonary oxygen concentrations (*E*). Multiple *t* tests using Holm-Sidak method were used. *P < 0.05, **P < 0.01, ***P < 0.001, and ****P < 0.0001. (*F*) Monocyte (*P* = 0.0277) and (*G*) neutrophil (*P* = 0.0031) counts were measured as ×10^3^/μl of ethylenediaminetetraacetic acid blood at Week 2 after infection; unpaired *t* test was used. (*H* and *I*) Week 3 postinfection plasma (*H*) IL-6 (*P* = 0.0452) and (*I*) IFN-γ (*P* = 0.0499) were measured by multiplex; unpaired *t* test was used. (*J*–*M*) Throughout the study, *Mtb* Erdman–infected nonhuman primates (NHPs) had higher bacterial burden in their BAL (*J*, not significant) that was higher at necropsy in (*K*) their lungs (*P* = 0.1404), (*L*) individual granulomas (*P* = 0.0001), and (*M*) liver (*P* = 0.0472) compared with *Mtb* CDC1551–infected NHPs; unpaired *t* tests were used. *P < 0.05, **P < 0.01, ***P < 0.001, and ****P < 0.0001. BPM = beats per minute; CRP = C-reactive protein; CXR = chest X-ray; Sp_O_2__ = oxygen saturation as measured by pulse oximetry.

Assessment of vaccine efficacy in the macaque model of TB has been performed by using either CDC1551 or Erdman as challenge strains (11–13, 27). We therefore performed a head-to-head comparison of virulence of the two strains in rhesus macaques. Fourteen rhesus macaques were infected with ∼200 deposited cfu of either *Mtb* CDC1551 (*n* = 8) or *Mtb* Erdman (*n* = 6) via aerosol. Survival was significantly reduced in NHPs infected with Erdman, with a maximum time to euthanasia of 7 weeks compared with a maximum of 11 weeks in those infected with CDC1551 (Figure 1A). Animals infected with Erdman had a significant and rapid increase in serum CRP concentrations (Figure 1B), and higher concentrations were detected in the sera of animals infected with Erdman than CDC1551 at endpoint (Figure E1A). At necropsy, animals challenged with Erdman had significantly higher thoracic radiograph scores in the lungs (Figure 1C). The radiograph results were consistent with clinical outcomes: macaques infected with *Mtb* Erdman exhibited greater respiratory distress (Figure 1D) and decreased blood oxygenation concentrations (Figure 1E) than those infected with *Mtb* CDC1551. At necropsy, fewer white blood cells were present in the peripheral blood of Erdman-infected than CDC1551-infected NHPs (Figure E1B). Early in infection, Erdman-infected macaques had a more exacerbated innate immune response than CDC1551-infected rhesus macaques. Erdman-infected NHPs had more basophils (Figure E1C), monocytes (Figure 1F), and neutrophils (Figure 1G) in their blood than CDC1551-infected NHPs. Throughout infection however, the two groups did not differ in blood lymphocyte concentrations (Figure E1D). The greater early influx of innate leukocytes into the periphery of animals infected with *Mtb* Erdman was supported by cytokine analysis. At Week 3 after infection, significantly elevated concentrations of proinflammatory cytokines IL-6 (Figure 1H) and IFN-γ (Figure 1I) were detected in the plasma of Erdman-infected rhesus macaques.

Differences in bacterial burden were apparent between the two groups starting atearly time points in the pulmonary compartment, with more bacilli recovered from BAL of animals infected with Erdman than CDC1551 between Weeks 3 and 7 (Figure 1J). One-log higher bacterial burden was found in the BAL of animals infected with Erdman than CDC1551 at endpoint (Figure E1E). The differences in bacterial burden between the two infection groups were not significant for the lung (Figure 1K), but necropsy was regularly scheduled for the animals according to the protocol at Week 12 after infection, and many of the CDC1551-infected animals survived the protocol duration and were necropsied at that point. Meanwhile, all Erdman-infected macaques required euthanasia by Week 7 (Figure 1A). It is possible that at Week 7, the differences in lung burdens would have been significant between the two groups. When bacterial burdens were measured in individual granulomas isolated from the two groups, a significant, almost 2-log higher *Mtb* load was identified in the animals infected with Erdman (Figure 1L). *Mtb* Erdman–infected NHPs possessed significantly more bacterial burden in the liver, indicating greater capacity to disseminate, relative to CDC1551 (Figure 1M). However, animals infected with CDC1551 harbored higher bacterial burdens than Erdman-infected animals in their bronchial lymph nodes (Figure E1F).

### Infection with Erdman Results in Greater Inflammatory Macrophage Response

**Figure 2.**
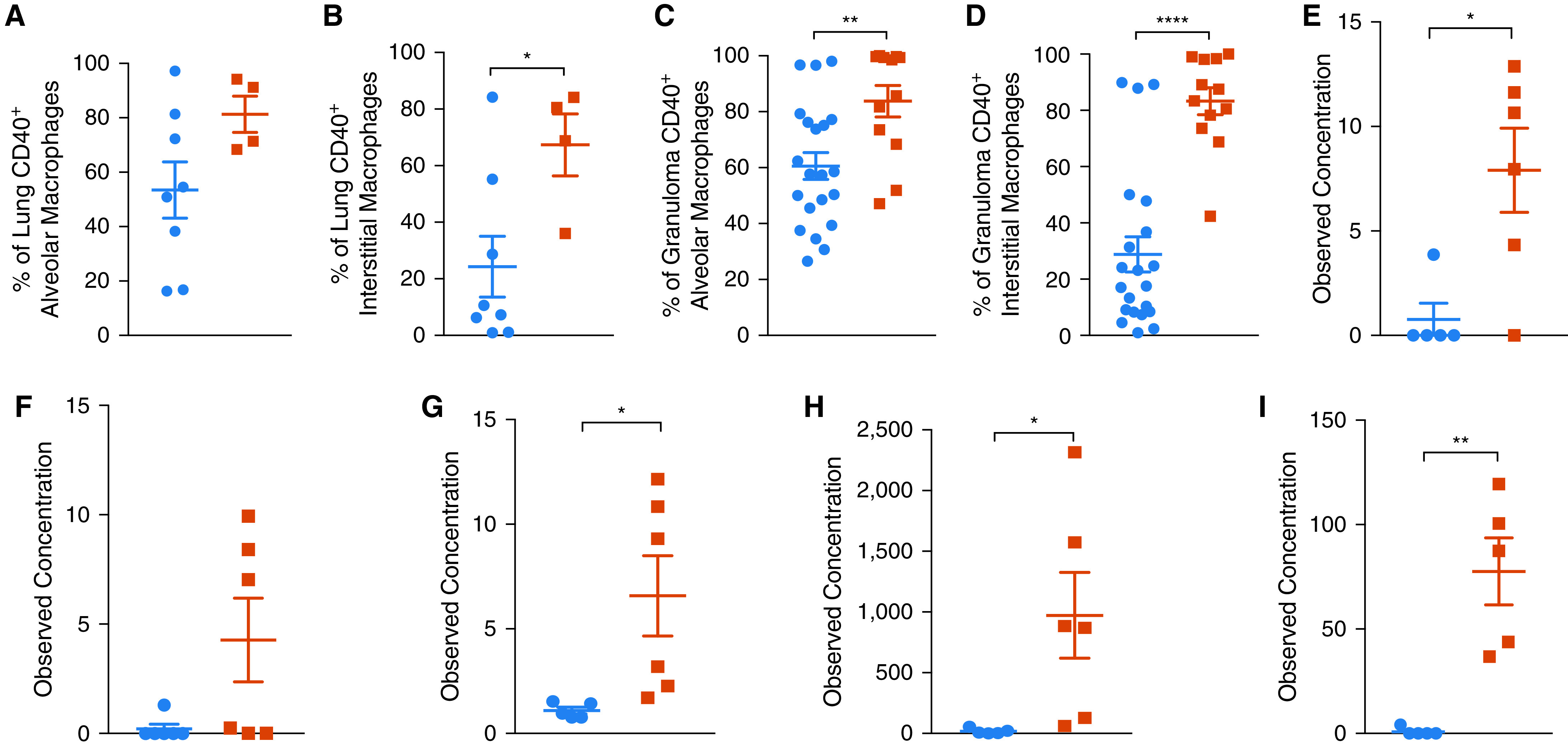
Activation phenotype in alveolar and interstitial macrophages after infection with comparative strains of *Mycobacterium tuberculosis* (*Mtb*) in the lungs of rhesus macaques. (*A*–*D*) In Erdman-infected nonhuman primates (NHPs) at necropsy, gross lung (*A*) alveolar (CD163^+^CD206^+^; *P* = 0.108) and (*B*) interstitial (CD163^−^CD206^+^; *P* = 0.0312) as well as (*C*) granuloma alveolar (*P* = 0.0048) and (*D*) interstitial (*P* < 0.0001) macrophages had significantly increased CD40 expression and activation. (*E* and *F*) At necropsy, Erdman-infected NHPs had more MIP1-α (macrophage inflammatory protein 1-alpha) in their (*E*) BAL supernatant (*P* = 0.0136) and (*F*) plasma (*P* = 0.0613). (*G*–*I*) As early as Week 3 after tuberculosis infection, Erdman-infected NHPs had significantly more (*G*) IL-1B (*P* = 0.0452), (*H*) I-TAC (*P* = 0.0368), and (*I*) IL-6 (*P* = 0.0014) in their BAL supernatant. Unpaired *t* tests were used. *P < 0.05, **P < 0.01, and ****P < 0.0001.

Inflammatory macrophage signaling in macaque lungs correlates with active TB disease (28). We therefore studied macrophage influx and signaling in the lungs of the two groups of animals exposed to CDC1551 and Erdman strains. There were no differences in the overall macrophage numbers, alveolar (Figures E2A–E2C) or interstitial (Figures E2D–E2F), in the BAL (Figures 2A and 2D), lungs (Figures E2B and E2E), or lung granulomas (Figures E2C and E2F) of the two groups of animals. The BAL in *Mtb* Erdman–infected NHPs, however, did reflect an early bias toward expansion of alveolar macrophages (Figure E2A), which shifted to support expansion of interstitial macrophages by Week 3 after *Mtb* infection (Figure E2D). The BAL samples from the Erdman-infected NHPs exhibited greater activation (cluster of differentiation 40 [CD40^+^]) of both alveolar and interstitial macrophages than the CDC1551 group over time (Figures E2G and E2H). Consistent with this, the lungs of animals infected with Erdman harbored activated (CD40^+^) alveolar (Figure 2A) and interstitial (Figure 2B) macrophages at the endpoint. This effect was even more pronounced in the granuloma tissue, where significantly higher concentrations of activated alveolar (Figure 2C) and interstitial (Figure 2D) macrophages were present in the Erdman group. Consistent with higher macrophage activation due to Erdman relative to CDC1551 infection, BAL and plasma concentrations of MIP1-α (macrophage inflammatory protein 1-alpha) also known as CCL3 (C-C motif ligand 3) were higher in the Erdman group at necropsy (Figures 2E and 2F). MIP1-α is one of the major factors produced by activated macrophages and monocytes (29) typically stimulated by IL-1β. The BAL concentrations of IL-1β were also significantly higher in the Erdman relative to the CDC1551 group at Week 3 after infection, confirming higher macrophage activation upon infection with this strain (Figure 2G). The main effect of MIP1-α expression from activated macrophages under the influence of IL-1β is the chemotaxis and activation of monocytes, neutrophils, basophils, and cells of the adaptive immune system. This is characterized by the increase in the concentrations of proinflammatory cytokines such as IL-6 and IFN-γ. We found that the BAL concentrations of IL-6 (Figure 2H) and interferon inducible T cell alpha chemoattractant (I-TAC) (C-X-C motif chemokine 11 [CXCL11], a key interferon-inducible T cell chemoattractant) (Figure 2I) were significantly higher in the Erdman- relative to the CDC1551-infected macaques at Week 3 after infection. I-TAC binds to its receptor C-X-C motif chemokine receptor 3 (CXCR3) with greater affinity than CXCL9 and CXCL10 and causes the recruitment of activated T cells (30, 31). Thus, increased activation of macrophages in Erdman-infected NHPs is a major driver of the pronounced peripheral inflammatory response detected by CRP elevation (Figure 1B) and cytokine measurements (Figures 1H and 1I) and could lead to an accelerated influx of T-helper cell type 1 responses to the lung.

### T Cell Characterization and Functionality

**Figure 3.**
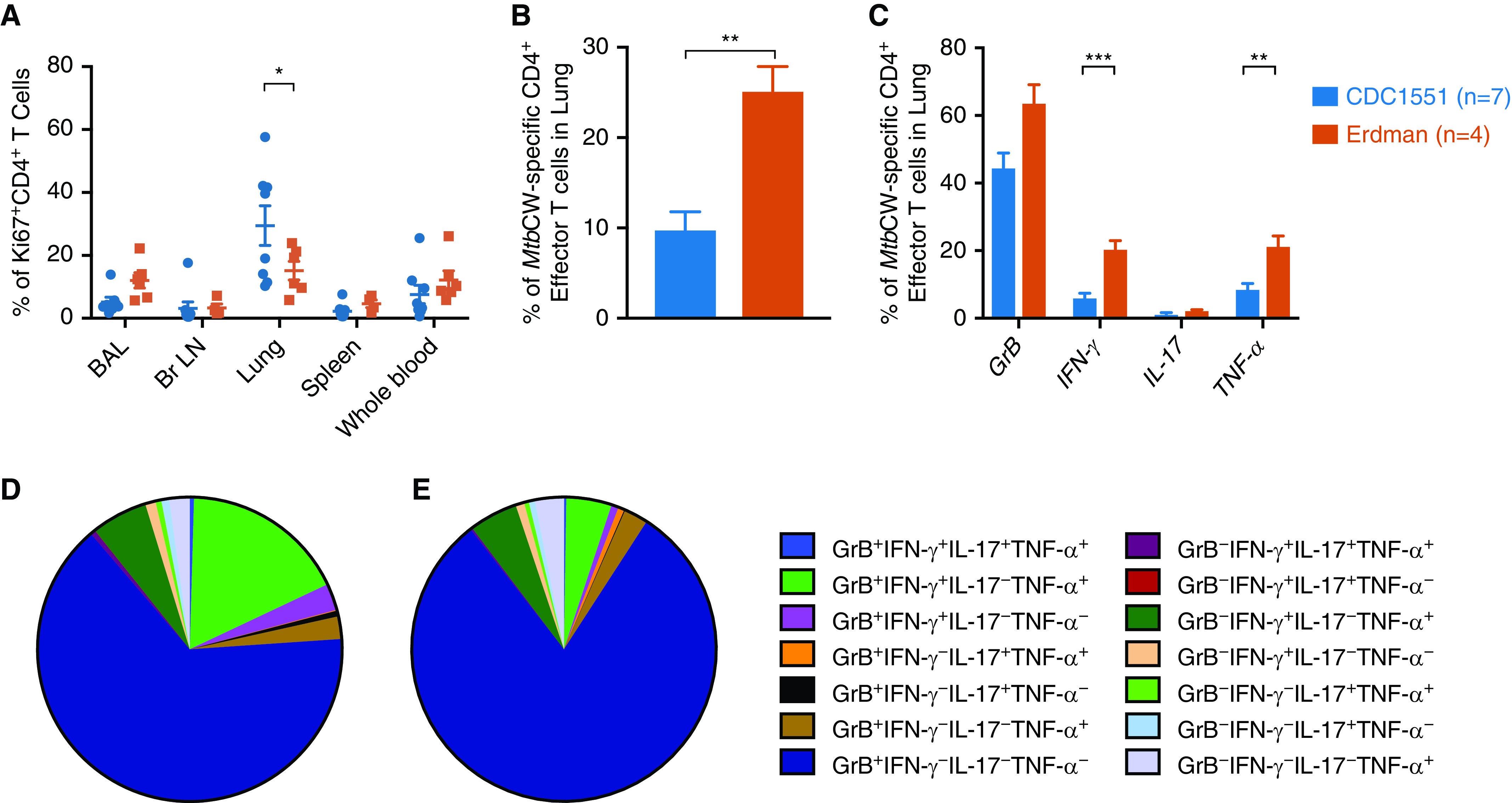
Lung T cells were more proliferative after CDC1551 infection in nonhuman primates (NHPs), but more antigen-specific effector T cells were observed after Erdman infection. (*A*) Proliferating CD4^+^ T cells were quantified by Ki67 expression in multiple tissues at necropsy. (*B*) Lung cells collected from Erdman-infected NHPs were stimulated overnight with *Mycobacterium tuberculosis* (*Mtb*) cell wall (*Mtb*CW) and produced significantly more cytokines than lung cells from CDC1551-infected NHPs when compared in bulk (*P* = 0.0013 unpaired *t* test) or (*C*) when IFN-γ (*P* < 0.0001) and TNF-α (tumor necrosis factor-α; *P* = 0.0033) were compared separately by multiple *t* tests. (*D* and *E*) When polyfunctional CD4^+^ T cell responses were considered, the repertoire of lung T cells producing cytokines in response to *Mtb* cell wall was more diverse in (*D*) Erdman- than in (*E*) CDC1551-infected rhesus macaques. Unpaired *t*-tests were used. *P < 0.05, **P < 0.01, and ***P < 0.001. Br LN = bronchial lymph node; GrB = granzyme B.

Throughout the course of the infection, CD4^+^ and CD8^+^ T cell concentrations in the peripheral blood (Figures E3A and E3B) and BAL (Figures E3C and E3D) were not different between the two infection groups. At necropsy, no differences were detected in the concentrations of CD4^+^ T cells in the blood, BAL, bronchial lymph node, and extrathoracic tissues, although the Erdman-infected animals harbored significantly more CD4^+^ T cells in the lungs (Figure E3E). At necropsy, CD8^+^ T cells in the tissues did not differ between NHPs infected with the two strains of *Mtb* (Figure E3F). More CD4^+^ (Figure E3G) and CD8^+^ (Figure E3H) T cells were, however, detected in the animals infected with CDC1551 in individual lung granulomas at endpoint. Differences were not observed in the expression of the chemokine receptors C-C chemokine receptor 5 (Figures E4A and E4B), CCR6 (Figures E4C and E4D), CCR7 (Figures E4E and E4F), or CXCR3 (Figures E4G and E4H) in the BAL, indicating that there were no variations in CD4^+^ or CD8^+^ T cell functional phenotypes between animals challenged with *Mtb* Erdman and CDC1551. Concentrations of human leukocyte antigen DR isotype (HLA-DR) and Ki67, used to mark T cell activation and proliferation, were also not significantly different in the two groups, although the concentrations of HLA-DR were higher in both the peripheral blood (Figures E5A and E5B) and BAL (Figures E5C and E5D) of the CDC1551-infected group. Throughout the course of infection, Ki67-associated proliferation increased but was matched in both CD4^+^ and CD8^+^ cells in the periphery (Figures E5E and E5F) and BAL (Figures E5G and E5H) of both groups of macaques. The percentage of Ki67^+^ CD4^+^ T cells present in the lung at the endpoint was significantly higher in the CDC1551-infected group despite lower bacterial burden, compared with the Erdman-infected group (Figure 3A). In the peripheral blood, however, *Mtb* Erdman–infected NHPs harbored higher potential for CD4^+^ (Figure E6A) and CD8^+^ (Figure E6B) effector T cell function and tissue retention at Weeks 1 and 3 after challenge, as indicated by CD69 expression. This trend was also reflected in the BAL at later time points (Figures E6C and E6D) and at necropsy in the general CD8^+^ T cell population (Figure E6E). The comparable T cell activation in early BAL may reflect delayed trafficking from the peripheral blood to the site of infection.

**Figure 4.**
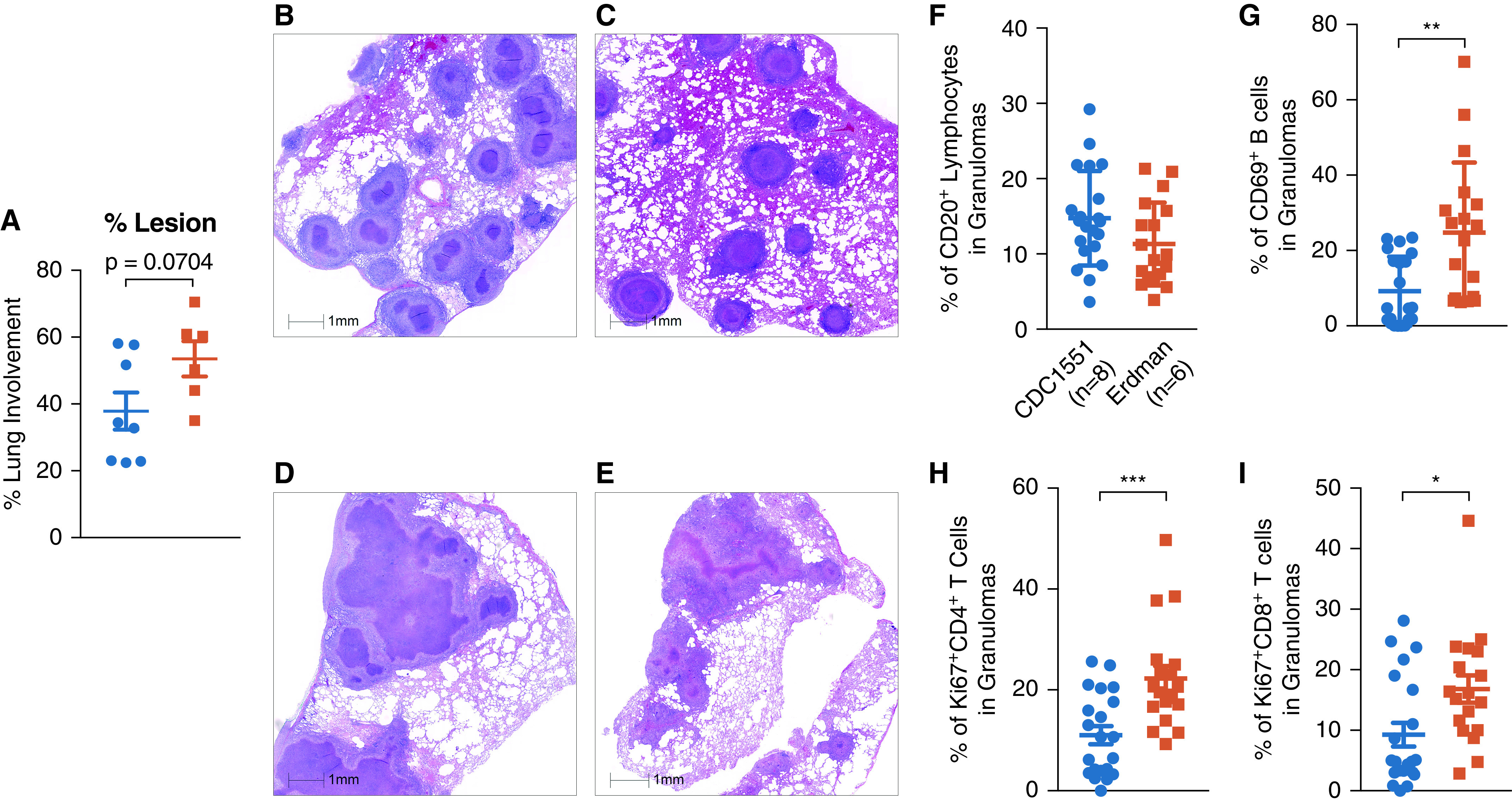
Comparable pathology was observed, but B cell activity was increased after *Mycobacterium tuberculosis* (*Mtb*) Erdman and CDC1551 infection in granulomas. (*A*) Percentage of lung involvement determined by board-certified veterinary pathologists (not significant; *P* = 0.0704). (*B* and *C*) Hematoxylin and eosin (H&E) representative figures show infected lung from CDC1551-infected nonhuman primate (NHP) necropsied at an early (*B*) and a late (*C*) time point. (*D* and *E*) H&E representative figures show infected lung from an Erdman-infected NHP necropsied at an early (*D*) and a late (*E*) time point. (*F*) B cells were quantified in granulomas as a proportion of lymphocytes. (*G*) Erdman-infected rhesus macaques had significantly more activation of their granuloma B cells with increased expression of CD69 (cluster of differentiation 69) (*P* = 0.0016). (*H* and *I*) Erdman-infected rhesus macaques had significantly more expression of proliferative marker Ki67 expressed on (*H*) CD4^+^ (*P* = 0.0006) and (*I*) CD8^+^ (*P* = 0.0154) T cells in their granulomas. This analysis compared up to three granulomas each from *n* = 6 Erdman- and *n* = 8 CDC1551-infected rhesus macaques. Unpaired *t* tests were used. Scale bars, 1 mm. *P < 0.05, **P < 0.01, and ***P < 0.001.

The ability for CD4 + effector T cells to produce TB antigen–specific cytokines was significantly increased at necropsy in the lungs of Erdman-infected NHPs, which experienced a significant increase in the proportion of CD4^+^ effector T cells that produced a cytokine, either IFN-γ, IL-17, or TNF-α, in response to stimulation with *Mtb* cell wall (Figure 3B). In response to *Mtb* cell wall, lung cells from Erdman-infected NHPs produced significantly more IFN-γ and TNF-α (Figure 3C). In addition, the proportion of CD4^+^ effector T cells capable of producing polyfunctional responses in T cells was significantly altered during challenge with different strains of *Mtb*. When lung cells from Erdman-infected animals (Figure 3D) were stimulated with peptides from *Mtb* cell wall, their CD4^+^ effector T cell fraction exhibited a significantly greater polyfunctional profile than samples from CDC1551-infected (Figure 3E) animals (Figure E7A). Thus, animals infected with *Mtb* Erdman (Figure 3D) possessed more CD4^+^ T cells that produced GrB (granzyme B) alone as well as GrB^+^IFN-γ^+^IL-17^−^TNF-α^+^ T cells than NHPs challenged with *Mtb* CDC1551 (Figures 3E and E7A). *Mtb* CDC1551–infected NHPs had significantly more non–cytokine-producing cells (GrB^−^IFN-γ^−^IL-17^−^TNF-α^−^ CD4^+^ T cells) than *Mtb* Erdman–infected animals (Figures 4C, 4D, and E7A). These results are consistent with the higher expression of CD69 in both the CD4^+^ and CD8^+^ T cell populations in the BAL of Erdman-infected macaques (Figures E6C and E6D), indicating that Erdman infection results in greater activation and *Mtb-*specific T cell responses. These results suggest that T cell responses established in the lungs of macaques infected with *Mtb* Erdman were characterized by greater activation as well as polyfunctionality than those infected with CDC1551, possibly because of increased amounts of *Mtb* antigens available at the infection site. Interestingly, the maximal potential of lung CD4^+^ effector T cells from CDC1551-infected NHPs to produce cytokines like IFN-γ, TNF-α, or IL-17, or cytolytic GrB, was not reduced as CD4^+^ effector T cells produced comparable concentrations of cytokines and chemokines as lung CD4^+^ effector T cells from Erdman-infected NHPs in response to phorbol 12-myristate 13-acetate and I stimulation (Figure E7B). Our results suggest that infection with *Mtb* Erdman might promote a localized hyperactive state of immune cells.

### Pathology

To investigate this further, we studied lung pathology in the two groups of animals in depth. In the lung tissue, as measured by a pathologist, increased lung involvement was observed in *Mtb* Erdman–infected NHPs, approaching significance (Figure 4A). Both groups of animals displayed extensive lung pathology, which was expected because of the nonphysiological high dose of infection. The pathology in *Mtb* CDC1551–infected animals was, however, represented by multiple individual circular lesions with well-defined inner myeloid and outer lymphocytic cellular layers, with limited central necrotic regions regardless of if the NHP was necropsied early because of clinical criteria (Figure 4B) or because of a scheduled endpoint (Figure 4C). However, the lesions in *Mtb* Erdman–infected animals appeared to be coalesced together with uncontrolled necrotic regions at both early and later necropsy time points (Figures 4D and 4E). Necrotic areas of TB granulomas are characterized by presence of hypoxia (32). It is therefore likely that infection with Erdman induces greater hypoxic response in the macaque lungs relative to CDC1551. Although the granulomas of Erdman-infected animals did not have more B cells (Figure 4F), they exhibited greater B cell activation (Figure 4G) and CD4^+^ (Figure 4H) and CD8^+^ (Figure 4I) T cell proliferation relative to the CDC1551-infected group. Coupled with the lower broad-spectrum, polyfunctional, antigen-specific cytokine production by T cells recruited to the lungs, and specifically granulomas of *Mtb* Erdman–infected macaques, the above pathology results suggest that the uncontrolled inflammatory macrophage activation and recruitment to the lungs led to dysregulated B and T cell activation consistent with TB disease (33).

### Gene Expression in the Lungs of Erdman- and CDC1551-Infected Animals

**Figure 5.**
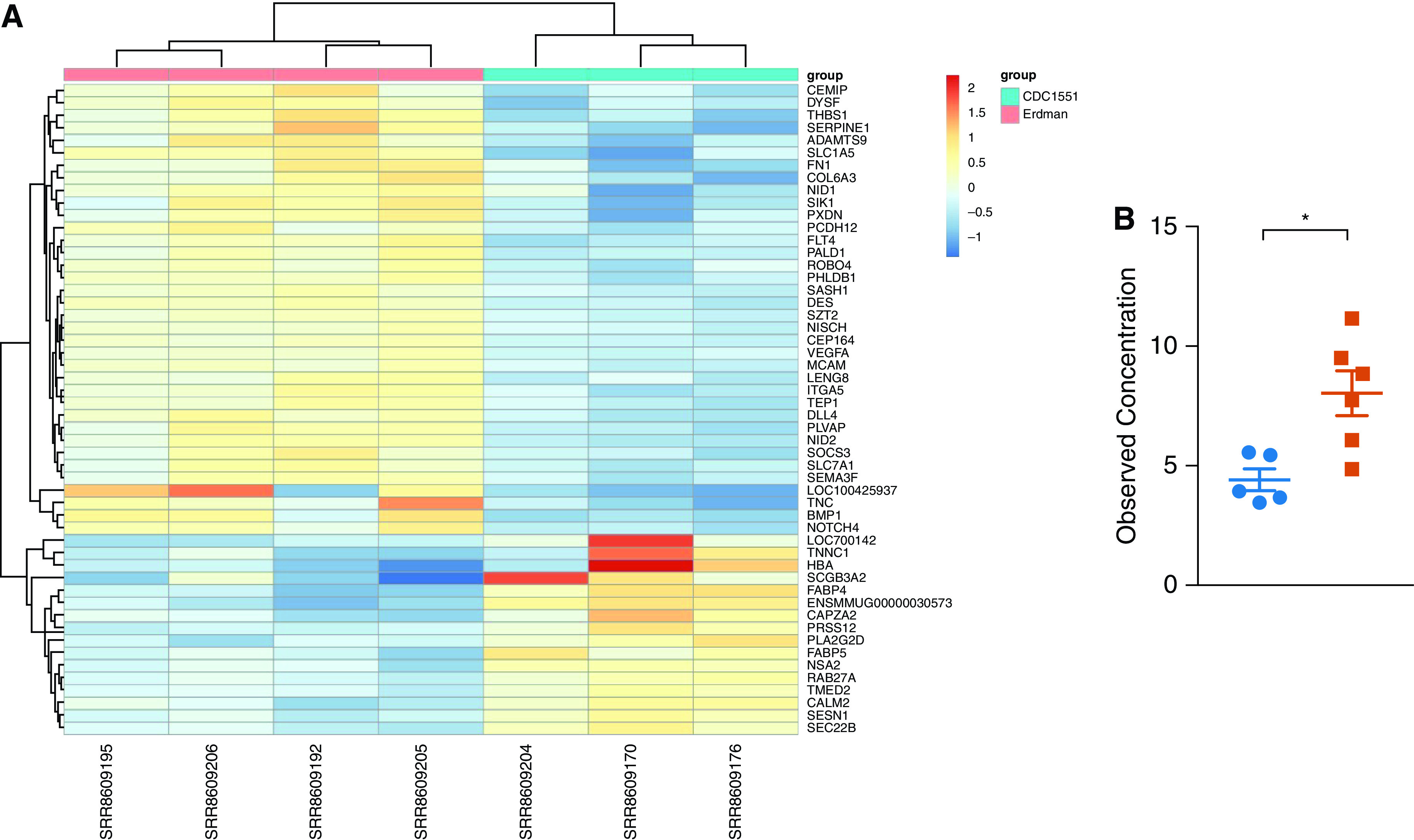
RNAseq gene-expression analysis revealed increased lung remodeling and T-helper cell type 2 (Th2) signaling in the lungs of Erdman-infected rhesus macaques. (*A*) RNAseq gene-expression analysis showing 52 differentially expressed genes were identified in the lungs of Erdman- versus CDC1551-infected macaques that revealed an increase in lung remodeling pathways, platelet-related genes, M2 and Th2 signaling (IL-4), and NOTCH4 (neurogenic locus notch homolog 4) signaling pathways. (*B*) Plasma IL-4 at Week 3 (*P* = 0.0102) measured by multiplex. Unpaired *t* tests were used. *P < 0.05.

To understand molecular signaling in the granulomas of macaques infected with the two strains, we performed RNAseq gene-expression analysis as described earlier (25). Fifty-two DEGs were identified in the lungs of Erdman- versus CDC1551-infected macaques (Figure 5A). Thirty-six genes were expressed to higher amounts in the lungs of animals infected with Erdman, with log fold change = 0.58–1.88. Sixteen genes were expressed to higher amounts in the lungs of macaques infected with CDC1551, with log fold change = 0.66–2.43 (Table 1). The number of DEGs was expectedly small because we were comparing two groups of animals with similar outcomes (progression to active TB), compared with our previous studies where we compared animals with distinct outcomes (active tuberculosis vs. latent tuberculosis infection [25]). The DEGs were used for downstream pathway analyses using Reactome pathway analysis (Tables 2 and 3). Four distinct pathways exhibited higher expression in the lungs of Erdman-infected animals:

1. Lung remodeling pathways (e.g., “Extracellular matrix organization,” “integrin cell surface interactions,” “degradation of ECM,” “ECM proteoglycans,” “Non-integrin membrane-ECM interactions,” and “Assembly of collagen fibrils and other multimeric structures”). The genes include BMP1, a positive regulator of TGF-β, MMP1, and MMP9, which have been associated with lung remodeling for granuloma formation (34, 35); extracellular matrix (ECM) protein Tenascin-C, which is expressed at high concentrations during lung organogenesis (36); SEMA3F, which plays a role in cell adhesion and migration in the lung (37); and PLVAP, which regulates basal permeability, leukocyte migration, and angiogenesis (38) (Table 2).
2. Platelet-related genes (e.g., “platelet degranulation,” “response to elevated platelet cytosolic Ca^2+^,” and “Platelet activation, signaling and aggregation”);
3. M2 and Th2 signaling (e.g., “IL-4 and IL-13 signaling” [e.g., SOCS3, which inhibits JAK and STAT3 (39)]); and
4. NOTCH4 (neurogenic locus notch homolog 4) signaling pathways “NOTCH4 Activation and Transmission of Signal to the Nucleus,” “Signaling by NOTCH4,” and “NOTCH4 Intracellular Domain Regulates Transcription.” Induced by response to hypoxia, NOTCH4 promotes undifferentiated cellular state. Important induced genes included DLL4, a hypoxia and vascular endothelial growth factor A (VEGFA) activated Notch ligand involved in angiogenesis by negatively regulating endothelial cell proliferation and migration and angiogenic sprouting (40); and VEGFA and NOTCH4, which together regulate cell fate specification, growth, differentiation, and patterning processes, mediating undifferentiated, necrotic, and profibrotic pathway in the lungs (41).

These results show that in lung lesions formed because of infection with Erdman, pathways related to ECM and tissue remodeling, hypoxia response, and in particular VEGFA and NOTCH signaling, were induced to higher amounts than during infection with CDC1551. These results support the observations of significantly more granulomatous pathology and in particular more necrotic regions in the granulomas of Erdman- than CDC1551-infected animals.

Genes that exhibited induced expression in the lungs of CDC1551- relative to Erdman-infected macaques were suggestive of greater antigen presentation (e.g., “Binding and Uptake of Ligands by Scavenger Receptors,” “Golgi-to-ER retrograde transport,” “ER to Golgi Anterograde Transport,” “Transport to the Golgi and subsequent modification,” “Intra-Golgi and retrograde Golgi-to-ER traffic,” and “COPII-mediated vesicle transport”). These results are consistent with the strong T cell responses mounted by CDC1551-infected macaques despite the presence of significantly lower *Mtb* antigen.

### Differential *In Vitro* Responses to Hypoxia by Erdman and CDC1551

**Figure 6.**
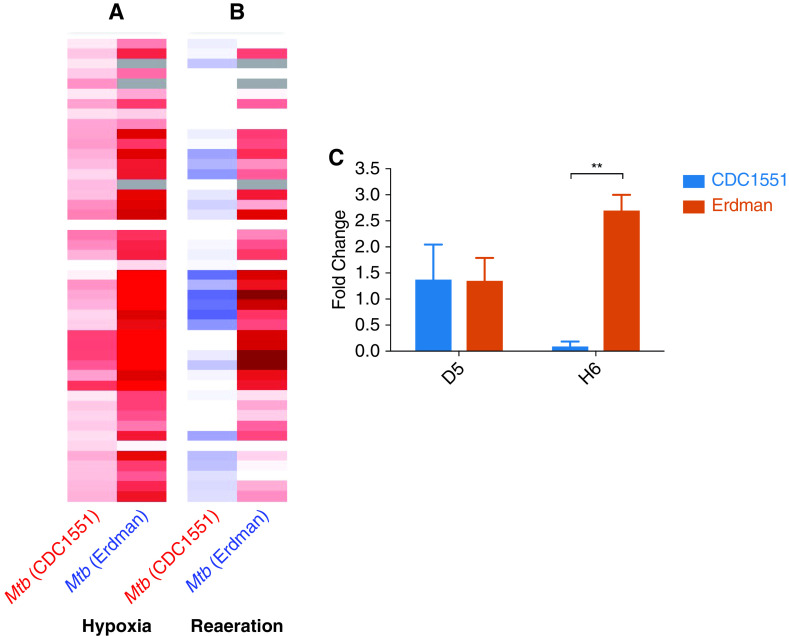
Differential patterns of hypoxia-related genes in *Mycobacterium tuberculosis* (*Mtb*) Erdman and CDC1551 were observed *in vitro* after hypoxia and reaeration. Bacteria were cultured under hypoxic conditions, and their responses were characterized by RNAseq. (*A* and *B*) Major differences were detected in the magnitude and kinetics of the expression of the DosR (dormancy survival regulator)-regulon, a set of ∼50 *Mtb* genes, which the pathogen uses to respond to hypoxia, (*A*) after hypoxia and (*B*) after the reaeration phase. (*C*) Quantitative real-time RT-PCR measuring the expression amounts of *dosR* in Erdman and CDC1551 samples at 5 days of hypoxia and at 6 hours of normoxia showed that the amounts of *dosR* were significantly higher in Erdman than in CDC1551 (*P* = 0.001). Unpaired *t* tests were used. **P < 0.01.

Erdman- rather than CDC1551-infected macaques harbored higher degrees of necrosis in the lungs, correlating with greater bacillary burdens and pathology in lungs. Necrosis is intricately linked to hypoxia, which is a key environmental cue that *Mtb* has evolved to respond to (32). Erdman-infected animals also expressed greater amounts of genes involved in response to hypoxia. We therefore measured the responses to hypoxia by the two strains *in vitro* using bacterial RNAseq. Major differences were detected in the magnitude and kinetics of the expression of the dormancy survival regulator (DosR)-regulon, a set of ∼50 *Mtb* genes that the pathogen uses to respond to hypoxia (Figures 6A and 6B). The expression of virtually all genes from the DosR-regulon, including sensor kinase DosS (42, 43) and response regulator DosR (32), was detected at significantly higher amounts in Erdman than in CDC1551 during hypoxia (Figure 6A). Even when *in vitro* hypoxia was terminated and normoxia restored (reaeration phase), the expression of the DosR-regulon gene members remained at much higher degrees in Erdman, whereas this expression rapidly normalized in CDC1551 (Figure 6B). To confirm these results, we performed quantitative real-time RT-PCR to measure the expression amounts of *dosR* in Erdman and CDC1551 samples at 5 days of hypoxia and at 6 hours of normoxia. We found that the amounts of *dosR* were significantly higher in Erdman than in CDC1551, confirming RNAseq results (Figure 6C).

## Discussion

Although a few studies have compared NHP responses to different strains of *Mtb*, focus has primarily been on characterizing pathological outcomes using radiology and not immune responses (10, 44). Here, consistent with results of strain–strain comparisons in murine models (6), we found that survival was significantly reduced, alongside greater activation of macrophages, in macaques infected with Erdman compared with those infected with CDC1551. We conclude that macrophage activation is one of the primary drivers of inflammation in TB disease, and this is supported by our recent working using single-cell approaches (28). Erdman-infected macaques exhibited less functional antigen-specific T cell responses, with significantly less cytokine production. Thus, increased inflammation and myeloid activation may promote excessive T and B cell activation in active TB, further promoting granulomatous pathology. Consistent with this, animals with high-dose Erdman infection exhibited greater pathology, with coalesced granulomas and more necrotic regions in these granulomas. Necrosis within granulomas is a characteristic of cell death and inflammation. Necrotic regions of the macaque granuloma core are characterized by greater hypoxia, and this allows for *Mtb* persistence (32). Consistent with this, we show greater induction of ECM and NOTCH4 pathways in the lungs of Erdman-infected macaques. NOTCH4 is induced by hypoxia, and ECM is critical for granuloma formation by lung remodeling, consistent with greater pathology in the Erdman group. Our results confirm the higher pathology in the Erdman group is associated with higher necrosis, greater response to hypoxia, greater macrophage activation, and increased *Mtb-*specific T cell responses. Our results show that the response to hypoxia mediates virulence in *Mtb*, with potential impacts on bacillary persistence, reactivation, and efficiency of therapeutics.
